# A multimodal MRI framework employing machine learning for detecting beginning cognitive impairment in Parkinson’s disease

**DOI:** 10.3389/fnins.2025.1689302

**Published:** 2025-11-26

**Authors:** Kevin Balßuweit, Peter Bublak, Kathrin Finke, Adriana L. Ruiz-Rizzo, Franziska Wagner, Carsten Klingner, Stefan Brodoehl

**Affiliations:** 1 Biomagnetic Center, Jena University Hospital, Friedrich Schiller University Jena, Jena, Germany; 2 Department of Neurology, Jena University Hospital, Friedrich Schiller University Jena, Jena, Germany; 3 Memory Center, Jena University Hospital, Jena, Germany

**Keywords:** cognitive impairment, Parkinson’s disease, machine learning, support vector machine, magnetic resonance imaging, functional neuroimaging, translational neuroscience

## Abstract

Cognitive deficits affect up to half of the patients with Parkinson’s disease (PD) within a decade of diagnosis, placing an increasing burden on patients, families and caregivers. Therefore, the development of strategies for their early detection is critical to enable timely intervention and management. This study aimed to classify cognitive performance in patients with PD using a binary support vector machine (SVM) model that integrates structural (high-resolution anatomical) and functional connectivity (FC; resting state) MRI data with clinical characteristics. We hypothesized that PD patients with beginning cognitive deficits can be detected through MRI in combination with machine learning. Data from 38 PD patients underwent extensive preprocessing, including large-scale FC and voxel-based analysis. Relevant features were selected using a bootstrapping approach and subsequently trained in an SVM model, with robustness ensured by 10-fold cross-validation. Although clinical parameters were considered during feature selection, the final best-performing model exclusively comprised imaging features—including gray matter volume (e.g., anterior cingulate gyrus, precuneus) and inter-network functional connectivity within the frontoparietal, default mode, and visual networks. This combined model achieved an accuracy of 94.7% and a ROC-AUC of 0.98. However, a model integrating clinical and only functional MRI data reached similar results with an accuracy of 94.7% and a ROC-AUC of 0.90. In conclusion, our findings demonstrate that applying machine learning to multimodal MRI data—integrating structural, functional, and clinical metrics—could advance the early detection of cognitive impairment in PD and could therefore be used to support timely diagnosis.

## 1 Introduction

Cognitive impairment is one of the most relevant non-motor symptoms in patients with Parkinson’s disease (PD), and their risk for developing dementia is up to six times higher than in the general population (Aarsland et al., 2001). Indeed, nearly half of PD patients develop dementia within 10 years of diagnosis (Williams-Gray et al., 2013), a progression that not only worsens quality of life and societal functioning but also places a substantial burden on caregivers (Leroi et al., 2012). Notably, the onset of dementia in PD is highly variable and is preceded by a state of mild cognitive impairment (MCI) (Yarnall et al., 2014; Aarsland et al., 2021). While MCI does not severely impair activities of daily living, it indicates a high risk for eventual dementia (Janvin et al., 2006; Hoogland et al., 2017).

Even in the absence of a cure, diagnosis of cognitive deficits in the MCI stage is crucial as it allows for the early initiation of symptomatic treatments to alleviate non-motor symptoms and to preserve cognition. Early identification of cognitive deficits can also allow for better outcome prediction, thereby supporting caregivers in planning and managing care effectively. Furthermore, it opens opportunities for participation in clinical trials that may lead to novel therapies (Ahlskog, 2011). However, the onset of cognitive deficits is insidious, and the course typically manifests as impairments in executive and visuospatial functions (Aarsland et al., 2021). Therefore, the initial MCI state often remains undetected in PD, underscoring the need for more sensitive and innovative diagnostic approaches to improve early identification (Weintraub et al., 2011; Melzer et al., 2012; Lopes et al., 2017; Wolters et al., 2019).

In this regard, advances in magnetic resonance imaging (MRI) bear a decisive potential. By revealing structural changes such as cortical atrophy and functional alterations like aberrant resting-state connectivity, they have facilitated comprehensive insights into the diagnosis, progression, and treatment of PD. However, similar to traditional clinical assessments, unimodal neuroimaging analyses may not fully capture the subtle changes associated with cognitive decline in PD.

Voxel-based morphometry (VBM) has become a widely adopted, automated technique for whole-brain analysis of structural MRI, allowing objective assessment of gray matter differences in vivo. It has largely supplanted earlier approaches based on manual region-of-interest (ROI) delineation, offering improved reproducibility and spatial coverage (Ashburner and Friston, 2001; Astrakas and Argyropoulou, 2010; Seyedi et al., 2020). In parallel, network-based analyses of resting-state fMRI data, which assign connectivity measures to predefined brain networks (Yeo et al., 2011), have provided transferable insights into brain function. Of note, integrating these two imaging modalities could leverage their complementary strengths and could therefore improve early detection of cognitive decline in PD.

Machine learning (ML) approaches have emerged as powerful tools in medical diagnostics. Supervised ML models such as support vector machines (SVM) are particularly adept at finding optimal boundaries in high-dimensional spaces, even with limited sample sizes (Jiang et al., 2017; Battineni et al., 2019). Studies have shown that SVM can efficiently differentiate patients with neurodegenerative disorders from controls, such as patients with Alzheimer’s disease, PD and Progressive Supranuclear Palsy (Magnin et al., 2009; Salvatore et al., 2014; Battineni et al., 2019). Moreover, studies employing SVM with MRI-derived features have reported good accuracy in identifying PD patients with cognitive impairment. For instance, structural MRI features have allowed achieving 81% accuracy (Peng et al., 2016). In another study, resting-state functional imaging data were analyzed with 80% accuracy, while a connectome analysis reveals a significant reduction in connectivity strength between PD patients with MCI and PD patients without cognitive deficits (Abós et al., 2017). Furthermore, structural imaging and clinical data were integrated into a preprint study for the prediction of cognitive decline with 78% accuracy (Smith et al., 2021).

While numerous studies have exploited either structural or functional MRI, to the best of our knowledge, only one multimodal approach that integrates both, along with further parameters, has yet been applied to identify PD patients with cognitive deficits (Zhu et al., 2024). Despite the relevance of using multimodal data (MRI, plasma or CSF markers), the use of routine data from non-invasive examinations ensures broader application outside of larger clinical facilities. In addition to MRI data, we also utilized simple clinical and demographic parameters. An association with cognitive deficits in PD was found for older age as well as a higher UPDRS and Hoehn and Yahr score (Guo et al., 2021).

The objective of this study was to develop a supervised ML framework for classifying PD patients according to their cognitive status, as defined by a validated MoCA threshold indicative of MCI, in the absence of manifest dementia. To ensure clinical applicability, we relied exclusively on clinically acquired MRI data—specifically, high-resolution structural and functional scans—without the need for specialized research protocols. Rather than assuming specific anatomical or network-level alterations a priori, our approach focused on data-driven feature selection from a broad set of imaging-derived and clinical parameters. We systematically compared three input configurations: (1) atlas-based gray matter volumes (GMV) with clinical variables, (2) functional connectivity (FC) metrics with clinical variables, and (3) a combined GMV+FC model, hypothesizing that integrating structural, functional, and clinical data would improve the accuracy of early cognitive impairment detection in PD.

## 2 Materials and methods

### 2.1 Subjects

Data from PD patients were taken from clinical records from the Department of Neurology between 2020 and 2024, Jena University Hospital, Germany. The diagnosis of PD was confirmed according to the International Parkinson and Movement Disorder Society (MDS) criteria (Postuma et al., 2015). All patients underwent a neuropsychological assessment with the Montreal Cognitive Assessment (MoCA) (Nasreddine et al., 2005) and an MRI examination comprising both high-resolution structural T1-weighted MRI and resting-state fMRI. Importantly, the interval between neuropsychological testing and MRI acquisition did not exceed 3 months. Exclusion criteria were applied to minimize confounding factors and included pre-existing severe cognitive deficits such as manifest dementia (MoCA score < 16), other relevant and active neurological, psychiatric, or systemic conditions (e.g., stroke, epilepsy, malignant disease, or movement disorders other than PD), as well as other factors potentially affecting cognitive performance (e.g., medication affecting consciousness) and intracranial surgery for treatment of PD.

A total of 118 patients with at least one MRI scan were retrospectively included, 46 of whom had both a T1-weighted and a complete resting-state fMRI scan. Although included in the imaging protocol, resting-state fMRI was sometimes not started or was terminated prematurely due to unspecified patient-related factors. The MRI scans were routine scans with a clinical focus, using an imaging protocol designed for the presence of neurodegeneration. Two scans were excluded after applying MRI quality checks (refer to the corresponding section).

Patients were divided into two groups based on their MoCA scores. As shown by a meta-analysis, a MoCA cutoff score of 22 and below provides greater diagnostic accuracy (86%) than the originally suggested cut-off score of 26 (78%) (Carson et al., 2018). Therefore, patients with a MoCA score of 24 or above were allocated to the group of PD patients with no cognitive deficits (PD-ND), while those with a score between 21 and 16 were assigned to the group of PD patients with cognitive deficits (PD-CD).

To ensure robust group separation, six patients with borderline scores (i.e., 22 or 23) were ruled out (described below). After applying these criteria, the final study cohort comprised 38 patients: 20 in the PD-CD group and 18 in the PD-ND group. Levodopa equivalent daily dose (LEDD) was calculated (Schade et al., 2020; Verber et al., 2020).

The study was approved by the local Ethics Committee of Jena University Hospital (approval number 2024-3229-BO). All clinical and imaging data were anonymized at the time of acquisition and stored in a secure institutional database prior to research use. No personally identifiable information was available to the investigators at any point. Reporting of this study adheres to the STROBE guidelines (von Elm et al., 2007).

### 2.2 MRI data acquisition

Neuroimaging data were acquired on a 3 T MR scanner (Trio, Siemens, Erlangen, Germany), including two complementary imaging sequences routinely used in clinical practice. High-resolution 3D T1-weighted Magnetization Prepared Rapid Gradient Echo (MPRAGE) scans were used for structural imaging (duration: approximately 5 min). These scans were acquired with 176 sagittal slices, each having an isotropic voxel size of 1.0 × 1.0 × 1.0 mm^3^. The T1-weighted sequence parameters were as follows: repetition time (TR) of 2,300 ms, echo time (TE) of 3.1 ms, inversion time (TI) of 1,100 ms, and a matrix size of 256 × 256 × 176.

Resting-state fMRI was performed using an echo-planar T2*-weighted sequence with a voxel size of 2.3 × 2.3 × 2.3 mm^3^ (duration: approximately 9 min). The fMRI acquisition parameters included a TR of 1,780 ms, a TE of 30 ms, and 72 trans-axial slices, with a matrix size of 94 × 94 × 72. A total of 203 image volumes were obtained. During the fMRI scan, patients were instructed to keep their eyes closed but to remain awake. Each image volume encompassed the entire cerebrum and cerebellum. Whenever possible, the fMRI sequences were recorded before the structural T1-weighted scans. In a few cases, the order of sequences was changed by the MRI technician for practical reasons, as noted during later data review; this was not systematically related to cognitive group or scan quality.

### 2.3 Data preprocessing

#### 2.3.1 MRI quality checks

Initially, MRI data were converted from DICOM to NIFTI format using SPM12 (Wellcome Department of Cognitive Neurology, London, UK; implemented in MATLAB R2023a, MathWorks, Natick, MA) (Penny et al., 2011). Quality assessment was then performed using CAT12 (computational anatomy toolbox) to automatically identify potential outliers based on established quality metrics (Gaser et al., 2024). In addition, all T1-weighted images and at least one resting-state fMRI image per subject were manually inspected to ensure that no distortions or artifacts were present. Only datasets that passed both the automated and manual quality checks were included in subsequent analyses.

#### 2.3.2 Structural data preprocessing: collection of gray matter volumes

For collecting gray matter volume (GMV), tissue segmentation was performed using the CAT12 toolbox. Standard segmentation pipelines were applied to partition the images into gray matter, white matter, and cerebrospinal fluid (CSF). The Neuromorphometrics atlas (Neuromorphometrics Inc., Somerville, MA, United States) was then employed in native space to delineate anatomical regions, and the corresponding GMV for each region was extracted. This process yielded 130 distinct regions per subject, and the resulting volumetric measurements served as features for subsequent SVM-based classification analyses. It is important to note that a full SPM-based VBM analysis was not performed, as this was beyond the scope of the present study.

#### 2.3.3 Resting-state fMRI: preprocessing and functional connectivity analysis

For resting-state fMRI preprocessing, the initial three volumes were discarded to mitigate MRI equilibration effects, and slice timing correction was applied to enhance temporal accuracy. Head motion was corrected via a rigid-body transformation aligning all functional images to the first volume, and the images were subsequently coregistered with the corresponding T1-weighted scans. These images were then normalized to Montreal Neurological Institute (MNI) space and smoothed using a 6-mm full-width-at-half-maximum (FWHM) Gaussian kernel to improve the signal-to-noise ratio. Nuisance variables from physiological and non-neuronal sources were regressed out, and a band-pass filter (0.01–0.1 Hz) was applied to retain neuronal frequencies while removing slow drifts and high-frequency noise (for further details, see Dahms et al., 2024).

For the FC analysis, voxel-wise time series were extracted and assigned to networks based on the Yeo2011 atlas (Yeo et al., 2011). The atlas divides the brain into 17 different large-scale networks, each of which corresponds to a different anatomical region of the brain and each of which can be assigned to exactly one of the following “superordinate” networks: frontoparietal network (FP-1, FP-2, FP-3, FP-4), default mode network (DMN-1, DMN-2, DMN-3), motor network (MOT-1, MOT-2, MOT-3), visual network (VIS-1, VIS-2), limbic network (LIM-1, LIM-2), dorsal attention network (DAN-1, DAN-2) and ventral attention network (VAN-1). The mean signal was computed for each network, and Pearson’s correlation coefficients were calculated for every network pair. After excluding autocorrelations and duplicate pairs, 136 unique FC values per subject were obtained. Finally, Fisher’s r-to-z transformation was applied to these coefficients, converting them into z-values that served as features for subsequent SVM-based classification analyses.

### 2.4 Machine learning pipeline

**FIGURE 1:**
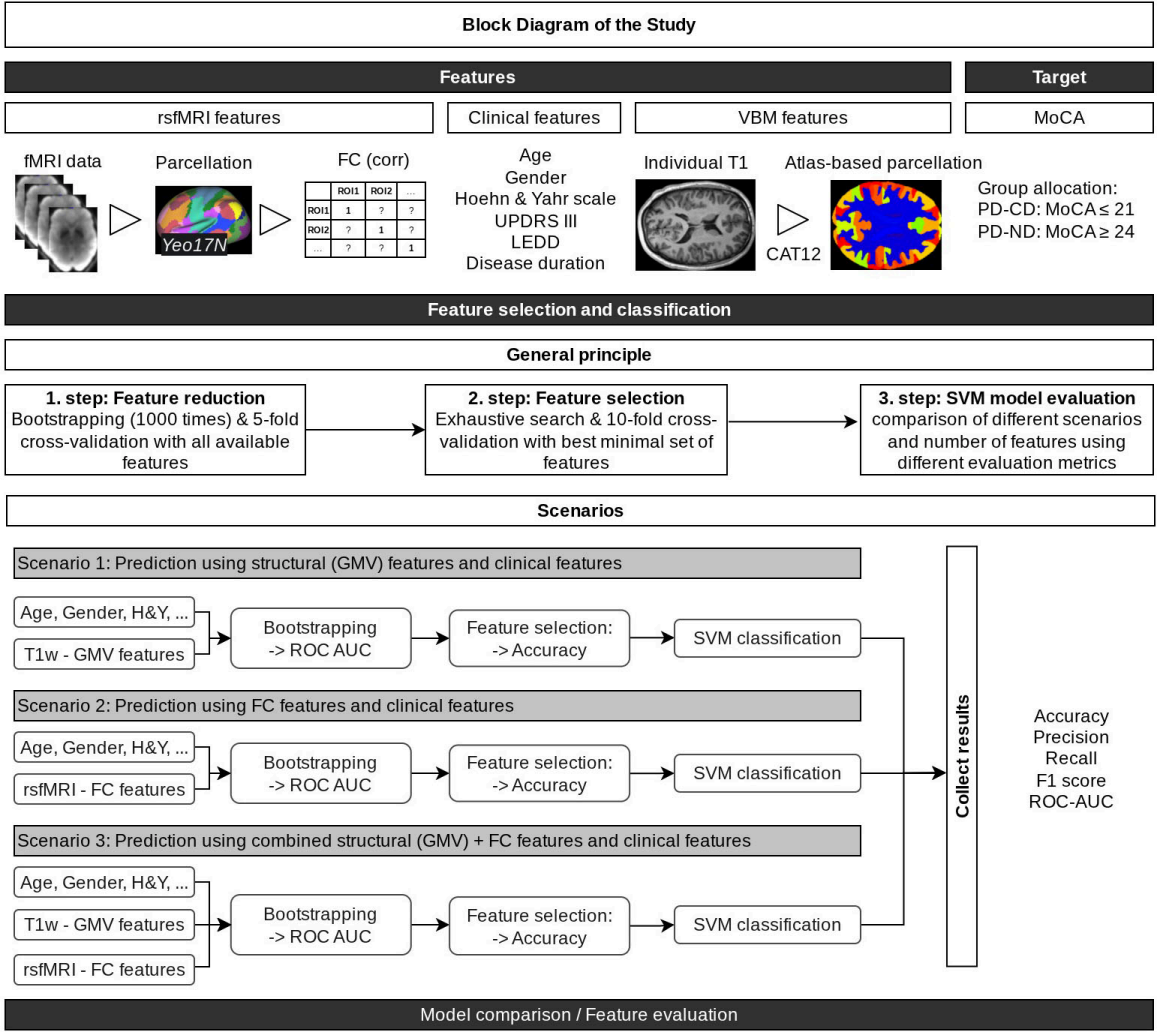
Overview of the machine learning pipeline. The pipeline integrates input features (clinical, GMV, and FC) and the target (MoCA group classification score) through sequential stages of feature selection, classification, and model evaluation. Time series data were extracted from all voxels of preprocessed resting-state fMRI volumes, then parcellated into 17 networks using the Yeo2011 atlas, and Pearson correlations were computed between network pairs. T1-weighted (MPRAGE) volumes were used to calculate gray matter volumes (GMV) for brain regions defined by the Neuromorphometrics atlas. Along with clinical data, all MRI features were z-score normalized, and three distinct feature sets were created (1. step: Feature reduction): one based on clinical data and solely GMV features, one on clinical data with and solely FC features, and one combining clinical data with both imaging modalities. For each scenario, bootstrapping with 1,000 iterations was applied to identify the 50 most consistently selected features (for details, see Methods—Feature Reduction). Feature selection (2. step) then identified the optimal combination of 1 to 6 features (i.e., the combination that yields the highest mean accuracy over 10 cross-validation loops), resulting in a total evaluation (3. step) of 18 models (6 feature combinations per scenario across 3 scenarios). FC, functional connectivity; H&Y, Hoehn and Yahr scale; LEDD, Levodopa equivalent daily dose; UPDRS III, Unified Parkinson’s Disease Rating Scale. Figure created with draw.io.

ML algorithms were implemented using the KNIME Analytics Platform (version 5.2.1), which has already been used in ML studies with fair results (Recenti et al., 2021; Amboni et al., 2022). For feature selection and model training, custom Python code (version 3.8.10) was employed via the open-source scikit-learn library (Pedregosa et al., 2018) integrated within KNIME. All features were z-score normalized using the KNIME normalizing node. Continuous clinical parameters (age, Hoehn and Yahr stage, UPDRS III, LEDD, and disease duration) were standardized to zero mean and unit variance, while the categorical variable *sex* was binary encoded (0 = female, 1 = male) before normalization. In line with our research question, we developed three distinct scenarios: (1) GMV features (with clinical data), (2) FC features (with clinical data), and (3) the combined GMV+FC feature set (with clinical data). An overview of our ML pipeline is provided in Figure 1.

#### 2.4.1 Feature reduction

Our feature reduction process started with a dataset containing 272 features, consisting of 130 atlas-based GMV values, 136 FC values, and 6 basic clinical parameters (age, sex, Hoehn & Yahr stage, UPDRS III score, levodopa equivalent daily dosage, and disease duration), along with a binary classifier indicating the presence or absence of cognitive deficits as defined by MoCA cutoffs. To identify features that consistently improved model performance, we implemented a bootstrapping algorithm with 1,000 iterations per bootstrap run.

In each bootstrap iteration, the algorithm began with an empty feature set and iteratively selected a random feature from the available pool. The performance of a linear SVM classifier was then evaluated using five-fold cross-validation, with the area under the receiver operating characteristic curve (ROC-AUC) serving as the performance metric. If the addition of a new feature increased the ROC-AUC, it was permanently added to the feature set and removed from the pool of unused features. This iterative process continued until no further improvements were observed or all features had been considered. After 1,000 bootstrap iterations, the frequency with which each feature was selected was aggregated to generate a ranking based on its consistency and contribution to model performance.

To address our research question, feature reduction was performed separately for the three above-mentioned scenarios. This approach enabled us to identify the most predictive features within each scenario for subsequent SVM-based classification analyses. The Python code implementing the algorithm within KNIME is provided in Supplementary section.

#### 2.4.2 SVM classification model development

For SVM classification, we performed an exhaustive search over all feature subsets drawn from the 50 highest-ranking candidates produced by each modality-specific pipeline. Subset evaluation was parallelized with *joblib* on a workstation equipped with 16 physical cores (32 logical threads), distributing the 10-fold cross-validation folds across threads and markedly reducing wall-clock time. Because the search space grows combinatorially—C(50, 6) ≈ 1.6 × 10^∧^7, whereas C(50, 7) ≈ 1.0 × 10^∧^8—we restricted the exhaustive search to subsets containing at most six features, the largest configuration tractable within the available computational budget.

For each subset size, we performed a grid search across kernel types (linear, RBF (radial basis function), and polynomial) and corresponding hyperparameters. The regularization parameter was tuned over C ∈ {0.01, 0.1, 1, 10, 100}; for RBF and polynomial kernels, γ ∈ {1e–3, 1e–2, 1e–1, “scale”}, and for polynomial kernels, degree ∈ {2, 3}. The optimal model was defined as the subset–hyperparameter combination that achieved the highest mean accuracy across repeated 10-fold cross-validation; precision, recall, F1-score and ROC-AUC were recorded as secondary metrics. This pipeline was executed independently for the three analytical scenarios, and in each case, the model with the highest mean accuracy was retained as the final classifier. The full implementation, including the parallelization code, is provided in Supplementary material.

The final, approximately balanced dataset of 20 PD-CD and 18 PD-ND cases required no resampling or class weighting for SVM training.

### 2.5 Model evaluation

Key evaluation metrics were computed to assess model performance and guide the selection of the optimal configuration. These metrics included accuracy, precision, recall, F1 score, and ROC-AUC. Accuracy was the ratio of correctly classified patients to the total number of patients, calculated as:


$$A\,c\,c\,u\,r\,a\,c\,y=\frac{\left(T\,P+T\,N\right)}{T\,P+T\,N+F\,P+F\,N}.$$

Precision (positive predictive value) was computed as:


$$P\,r\,e\,c\,i\,s\,i\,o\,n=\frac{T\,P}{T\,P+F\,P}.$$

Recall (sensitivity) was calculated as:


$$R\,e\,c\,a\,l\,l=\frac{T\,P}{T\,P+F\,N}.$$

The F1 score, representing the harmonic mean of precision and recall, was determined by the equation:


$$F\,1\,s\,c\,o\,r\,e=2\times \frac{P\,r\,e\,c\,i\,s\,i\,o\,n\times R\,e\,c\,a\,l\,l}{P\,r\,e\,c\,i\,s\,i\,o\,n+R\,e\,c\,a\,l\,l}.$$

In these definitions, true positives (TP) denote the number of patients classified as PD-CD by both the MoCA and the model; true negatives (TN) are those classified as PD-ND by both; false positives (FP) are patients classified as PD-ND by the MoCA but as PD-CD by the model; and false negatives (FN) are patients classified as PD-ND by the model but as PD-CD by the MoCA.

### 2.6 Inter-group comparison of MRI features

To test whether any single MRI feature shows a covariate-adjusted group effect, we supplemented the multivariate SVM pipeline with a univariate analysis of covariance (ANCOVA). Age and sex—and, for GMV metrics, total intracranial volume (TIV)—were included as covariates so that the resulting F-statistics capture variance attributable uniquely to cognitive status. This classical analysis provides an effect-size benchmark for the ML-selected features and conforms to current neuroimaging reporting guidelines.

Statistical analyses including ANCOVA tests were run in JASP v0.18.1 (Department of Psychological Methods, University of Amsterdam) (Love et al., 2019).

## 3 Results

### 3.1 Demographics and clinical characteristics

Detailed demographic and clinical statistics of the study cohort are shown in Table 1.

**TABLE 1:** Sociodemographic and clinical information by group.

*n* = 38	Range	PD-CD (n = 20)	PD-ND (*n* = 18)	*p*-value
Age, y, mean (SD)	[57, 79]	68.75 (6.56)	65.39 (6.17)	0.113 (†)
Gender (m/f)	–	9/11	9/9	0.758 (χ)
Formal education (≤ 12y/ > 12y)	–	2/18	1/17	0.612 (χ)
UPDRS III, score, mean (SD)	[7, 40]	18.90 (7.33)	20.11 (8.48)	0.640 (†)
H&Y stage (1:1.5:2:2.5:3:4)	–	1:2:4:2:7:4	2:2:4:3:7:0	0.435 (χ)
Disease duration, y, mean (SD)	[1, 19]	5.70 (6.58)	5.11 (4.14)	0.741 (‡)
Subtype (E:AR:TD)	–	9:8:3	4:11:3	0.362 (χ)
Emphasized side (L/r)	–	8/12	8/10	0.782 (χ)
LEDD, mg/d, mean (SD)	[52, 2282]	640.05 (542.97)	605.34 (345.94)	0.818 (†)
MoCA, score, mean (SD)	[16, 29]	19.30 (1.69)	25.83 (1.62)	<0.001* (†)

For continuous variables, standard deviations (SD) are shown in parentheses. Disease duration is described by the time since initial diagnosis. PD-CD, Parkinson’s disease patients with cognitive impairment; PD-ND, Parkinson’s disease patients without cognitive deficits; y, years; UPDRS, Unified Parkinson’s disease rating scale III (motor examination); H&Y, Hoehn and Yahr scale; E, Equivalent motor subtype; AR, Akinetic/rigid motor subtype; TD, Tremor-dominant motor subtype; LEDD,, levodopa equivalent daily dose; MoCA, Montreal cognitive assessment. † Independent sample *t*-test (for continuous variables with normal distribution). ‡Welch test (for continuous variables with abnormal distribution). χChi-squared test (χ^2^, for categorical variables).

*Represents a significant increase between groups.

### 3.2 Exhaustive feature selection and model optimization

The feature sets resulting from bootstrapping-based feature reduction for each scenario are presented in Supplementary Tables 1–3 and served as the basis for subsequent exhaustive feature selection. Using the 50 most predictive features from each modality-specific scenario, we conducted an exhaustive search to identify the optimal feature subsets for each scenario. The best-performing feature combinations and corresponding performance metrics are summarized in Table 2.

**TABLE 2:** SVM model performance comparison across feature subset sizes and modalities.

	GMV		FC		GMV + FC	
*n*	Accuracy	ROC-AUC	Accuracy	ROC-AUC	Accuracy	ROC-AUC
1	60.8%	0.61	68.3%	0.64	65.8%	0.64
2	73.6%	0.75	78.6%	0.83	78.9%	0.83
3	81.4%	0.85	84.2%	0.86	84.2%	0.86
4	86.9%	0.82	89.4%	0.88	91.9%	0.88
5	90.0%	0.85	89.7%	0.87	91.9%	0.87
6	92.2%	0.86	94.7%	0.90	94.7%	0.98

GMV, gray matter volume; FC, functional connectivity, ROC AUC, area under the receiver operating characteristic curve.

#### 3.2.1 GMV-based scenario (with clinical data)

Table 3 shows the feature combinations and corresponding performance metrics for GMV-based SVM models using different subset sizes (1–6 features). With only one feature (left superior temporal gyrus), the model achieved an accuracy of 60.8% and a ROC-AUC of 0.61. Increasing the number of features consistently improved performance. In particular, the 6-feature model (third ventricle, right angular gyrus, left anterior cingulate gyrus, right precuneus, left superior frontal gyrus and right medial orbital gyrus) yielded the highest accuracy of 92.2%, together with a precision of 93.3%, a recall of 92.5%, an F1 score of 0.92 and a ROC-AUC of 0.86.

**TABLE 3:** Selected features using GMV and clinical data.

*n*	Features	Accuracy	Precisio*n*	Recall	F1 score	ROC-AUC
1	Left superior temporal gyrus	60.8	54.3	59.4	0.54	0.61
2	Left precentral gyrus, Right precuneus	73.6	75.8	72.5	0.72	0.75
3	3rd Ventricle, Right MPrG, Right cuneus	81.4	83.1	81.8	0.81	0.85
4	3rd Ventricle, Right cuneus, Right PCgG, Right MPoG	86.9	88.7	86.9	0.87	0.82
5	Left ACgG, Hoehn & Yahr scale, Right precuneus, Left precuneus, Right ACgG	90.0	90.0	90.0	0.90	0.85
6	3rd Ventricle, Right AnG, Left ACgG, Right precuneus, Left SFG, Right MOrG	92.2	93.3	92.5	0.92	0.86

Each metric is expressed as the mean value over 10 cross-validation repeats. MPrG, precentral gyrus medial segment; PCgG, posterior cingulate gyrus; MPoG, postcentral gyrus medial segment; ACgG, anterior cingulate gyrus; AnG, angular gyrus; SFG, superior frontal gyrus; MOrG, medial orbital gyrus.

#### 3.2.2 FC-Based SVM models (with clinical data)

Table 4 lists the best-performing feature subsets and metrics for FC-based classification, ranging from 1 to 6 features. A single feature (DMN-2∼FP-4) gives an accuracy of 68.3%, which increases steadily as the number of features increases. The 6-feature model (DMN-3–VIS-2, FP-4–MOT-1, Age, DAN-2–DMN-1, DAN-2–FP-4, DMN-3–MOT-1) achieves the best performance with an accuracy of 94.7% and a ROC-AUC of 0.90, performing better than the GMV-only models.

**TABLE 4:** Selected features using FC and clinical data.

*n*	Features	Accuracy	Precision	Recall	F1 score	ROC-AUC
1	DMN-2∼FP-4	68.3	68.5	68.1	0.68	0.64
2	DMN-2∼VIS-2, FP-4∼MOT-1	78.6	79.6	78.1	0.78	0.83
3	DMN-2∼VIS-2, FP-4∼MOT-1, DAN-2∼DMN-1	84.2	85.2	83.8	0.84	0.86
4	FP-3∼FP-4, FP-2∼LIM-2, FP-2∼VIS-1, Age	89.4	90.8	90.0	0.89	0.88
5	FP-3∼FP-4, FP-2∼LIM-2, FP-2∼VIS-1, Age, LIM-1∼VAN-1	89.7	90.4	90.0	0.90	0.87
6	DMN-3∼VIS-2, FP-4∼MOT-1, Age, DAN-2∼DMN-1, DAN-2∼FP-4, DMN-3∼MOT-1	94.7	95.4	95.0	0.95	0.90

Each metric is expressed as the mean value over 10 cross-validation repeats. DMN, default mode network; FP, frontoparietal network; VIS, visual network; MOT, motor network; DAN, dorsal attention network; LIM, limbic network; VAN, ventral attention network.

#### 3.2.3 Selected feature sets: combined GMV and FC features (with clinical data)

In the integrated approach, top-ranking GMV and FC features, along with clinical data, were merged into a single pipeline. As shown in Table 5, accuracy steadily increased from 65.8% with a single feature (DMN-2–VIS-2) to 94.7% for a 6-feature subset (DMN-3–VIS-2, FP-4–MOT-1, DMN-2–VIS-2, left anterior cingulate gyrus, left precentral gyrus, and right calcarine cortex). The final model achieved an F1 score of 0.95 and a ROC-AUC of 0.98.

**TABLE 5:** Selected features using GMV, FC and clinical data.

*n*	Features	Accuracy	Precision	Recall	F1 score	ROC-AUC
1	DMN-2∼VIS-2	65.8	71.8	65.6	0.64	0.64
2	FP-3∼FP-4, Right Putamen	78.9	78.8	78.8	0.79	0.83
3	FP-3∼FP-4, LIM-1∼LIM-2, Left posterior insula	84.2	86.2	84.4	0.84	0.86
4	FP-3∼FP-4, FP-3∼VIS-2, 3rd Ventricle, Left posterior insula	91.9	93.8	92.5	0.92	0.88
5	FP-3∼FP-4, Age, Left ACgG, Right cuneus, Left LOrG	91.9	93.3	91.9	0.92	0.87
6	DMN-3∼VIS-2, FP-4∼MOT-1, DMN-2∼VIS-2, Left ACgG, Left PrG, Right Calc	94.7	95.4	95.0	0.95	0.98

Each metric is expressed as the mean value over 10 cross-validation repeats. DMN, default mode network; VIS, visual network; FP, frontoparietal network; LIM, limbic network; ACgG, anterior cingulate gyrus; LOrG, lateral orbital gyrus; MOT, motor network; PrG, precentral gyrus; Calc, calcarine cortex.

**FIGURE 2:**
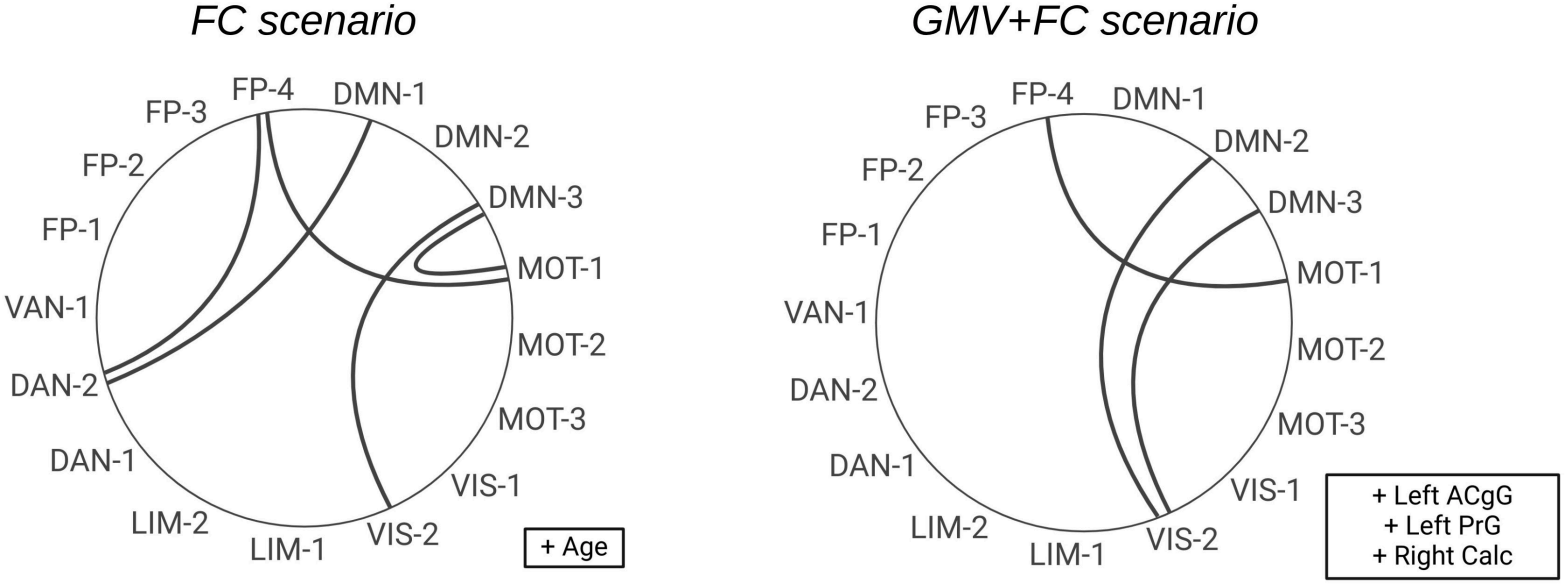
Circular diagrams illustrating the selected FC features for *n* = 6 features. On the left side, the features from the second scenario are shown (clinical and FC features). The features from the third scenario are displayed on the right side (clinical, GMV and FC features). The clinical and GMV features are shown in boxes to the right of the respective diagram. ACgG, anterior cingulate gyrus; PrG, precentral gyrus; Calc, calcarine cortex; DMN, default mode network; FP, frontoparietal network; VIS, visual network; MOT, motor network; DAN, dorsal attention network; LIM, limbic network; VAN, ventral attention network.

Figure 2 provides a visual overview of the top-ranked FC and clinical features selected for the 6-feature models in the scenario using FC and clinical data (left circle) and the combined scenario applying GMV, FC and clinical data (right circle). Clinical and GMV features, such as age, left anterior cingulate gyrus (ACgG) and left precentral gyrus (PrG), are shown in boxes adjacent to each graph. This presentation highlights the key network features (e.g., between the default mode network, the frontoparietal network, the motor and the visual network) that most effectively discriminate between PD-CD and PD-ND.

Across all scenarios, the best-performing models consistently employed a linear kernel with C = 1, while RBF and polynomial kernels did not further improve classification performance.

### 3.3 Intergroup comparison of MRI features—ANCOVA analysis

The complete data matrix comprised 266 MRI features per subject, including 136 FC features and 130 GMV features. To isolate the impact of cognitive status on brain structure and connectivity, an ANCOVA analysis was performed to compare MRI features between the PD-CD and the PD-ND group. The detailed ANCOVA results for the features selected by the SVM are listed in Supplementary Table 4. Differences in GMV and FC did not survive control for multiple comparisons via the Benjamini-Hochberg method (Benjamini and Hochberg, 1995). FDR-corrected values are also presented in Supplementary Table 4.

#### 3.3.1 Gray matter volume

Overall GMV did not differ significantly between the PD-CD and PD-ND groups. However, a few regional differences were observed. Specifically, compared to the PD-ND group, the PD-CD group showed slightly higher GMV in the left ACgG (*F* = 4.345, *p* = 0.045, uncorrected), whereas the volume in the optic chiasm was found to be slightly lower in the PD-CD group (*F* = 4.215, *p* = 0.048, uncorrected).

#### 3.3.2 Functional connectivity values

Analysis of the FC data revealed significant intergroup differences. Within the frontoparietal network (FPN), FC between FP-1 and FP-2 was significantly increased in the PD-CD compared to the PD-ND group (*F* = 5.942, *p* = 0.020, uncorrected), as was the FC between FP-3 and FP-4 (*F* = 5.505, *p* = 0.025, uncorrected). Furthermore, FC between the default mode network (DMN) and the visual network was significantly increased in the PD-CD group compared to the PD-ND group for both DMN-2–VIS-2 (*F* = 4.789, *p* = 0.036, uncorrected) and DMN-3–VIS-2 (*F* = 4.166, *p* = 0.049, uncorrected). Additionally, a near-significant reduction in FC between FP-2 and VIS-1 (*F* = 4.140, *p* = 0.050, uncorrected) indicated a trend toward diminished FP–visual network FC in PD-CD relative to PD-ND patients.

## 4 Discussion

### 4.1 Key findings and contributions

In this study, we developed a multimodal ML model that integrated structural (gray matter volume, GMV) and functional (resting-state functional connectivity, FC) MRI data with minimal clinical parameters to distinguish PD patients with beginning cognitive deficits (PD-CD) from those with normal cognition (PD-ND). Using only routine hospital-grade T1-weighted and resting-state scans, the model achieved promising classification results. We employed extensive image processing and model building, namely a bootstrapping technique followed by an exhaustive feature search and 10-fold cross-validation to identify a compact and highly discriminative feature set. Notably, a six-feature model attained a classification accuracy of 94.7% and ROC-AUC of 0.98, highlighting a parsimonious yet powerful biomarker signature.

The models benefited from key GMV measures (notably in the anterior cingulate gyrus and precuneus) and inter-network FC metrics. Models based solely on GMV showed lower performance, whereas resting-state FC features—particularly those reflecting changes in the frontoparietal (FPN) and default mode network (DMN) with other networks—were especially informative. These network-level changes point to a reorganization of functional brain architecture in PD-CD patients and underscore the importance of network disruption in the pathophysiology of cognitive decline. Importantly, combining imaging modalities with robust clinical markers, such as age and Hoehn and Yahr stage, further improved predictive power in models with fewer features.

### 4.2 Model performance and methodological considerations

Recent ML studies in neurodegenerative diseases have predominantly relied on MRI data, particularly structural and functional modalities (Altham et al., 2024). A significant way to use structural MRI is VBM analysis to quantify GMV and identify regional atrophy patterns (Smith et al., 2021; Suo et al., 2021; Beheshti and Ko, 2024). fMRI provides insights into FC, highlighting network disruptions associated with cognitive impairment to be detected by whole-brain connectome analyses and ML (Abós et al., 2017; Ysbæk-Nielsen, 2024). Zhu et al. showed that the brain changes detected by RS-MRI can better detect cognitive deficits than T1-MRI and reached mean accuracy of 76.2% and a 0.84 ROC-AUC (Zhu et al., 2024). In our study, we identified an optimal set of six features derived from clinical, structural and functional MRI data that discriminated between PD patients with and without mild cognitive deficits with high accuracy, precision and sensitivity (recall). We found more prominent differences in FC compared to GMV, with the classification performance of the corresponding scenario being slightly better, regardless of the number of features. However, we used different approaches for image preprocessing, e.g. large-scale FC analysis to assess connectivity at the network level.

The classification model was embedded within a 10-fold cross-validation framework, in which the dataset was divided into 10 approximately equal parts. In each fold, one part was used as the test set while the remaining nine parts served as the training data, and the final performance was obtained by averaging across all folds. To select the most informative features, exhaustive feature selection was performed. In this iterative procedure, all possible feature combinations were evaluated for subset sizes ranging from 1 to 6. Given that choosing 6 features from a total of 50 results in a combinatorial explosion (e.g., over 11 million possible subsets), the evaluation process was parallelized to leverage all available CPU cores, thereby significantly reducing computation time—a strategy in the spirit of recent methods (Lausser et al., 2022). This approach enabled the identification of feature combinations that exploit complementary information across modalities. However, it should be noted that exhaustive search alongside cross-validation carries the risk of data leakage.

Another important issue is the challenge of feature reduction. For example, Álvarez et al. employed multiple algorithms, including wrapper and filter methods, to reduce FDG-PET-derived features for the diagnosis of neurodegenerative diseases, achieving a reduced feature set without compromising performance (Álvarez et al., 2019). Similarly, Tang et al. reviewed various dimensionality reduction methods for brain imaging data, noting that while linear approaches such as principal component analysis (PCA) are computationally efficient, they may miss nonlinear relationships that more refined techniques can capture (Tang et al., 2021). In our study, we implemented a bootstrapping approach for each scenario separately to reduce the original set of input features to the 50 most consistently selected, thereby preserving the covariance structure between clinical, GMV and FC measures. Similar bootstrapping-based strategies have been shown to improve robustness and reproducibility in high-dimensional ML tasks (Abeel et al., 2010). Our SVM model used these features to find the optimum combination for classification. While classification accuracy increases continuously with the number of features used in all scenarios, we applied a parsimonious final model with few discriminative features.

### 4.3 Neurobiological interpretation

Considering selected GMV features across all numbers (1–6) of features, several selected features reflect parts of the DMN (precuneus, posterior cingulate cortex, angular gyrus), primary motor and somatosensory cortices, as well as areas that are part of the visual (cuneus, calcarine cortex) and auditory (left superior temporal gyrus) systems. The third ventricle is often selected and serves as a nonspecific atrophy marker. Third ventricle enlargement—frequently selected within our feature set—is widely recognized as a non-specific indicator of central brain atrophy, often reflecting degeneration of deep gray structures such as the thalamus (Guenter et al., 2022). Moreover, in PD, third ventricle enlargement has been linked to early cognitive impairment in PD-MCI cohorts (Dalaker et al., 2011). The left anterior cingulate gyrus (ACgG) emerged consistently as a stable predictive feature across both the FC-only and combined GMV + FC scenarios. This is consistent with existing MRI literature, which has demonstrated early structural involvement of the anterior cingulate cortex in PD-MCI patients who later developed dementia (Compta et al., 2013; Hou and Shang, 2022). The integration of Hoehn and Yahr stage and age (clinical data+GMV+FC scenario) indicates the relevance of clinical markers for the diagnosis. Notably, in the combined model including all six features, classical clinical parameters were not selected. These findings suggest that age-related neurodegenerative changes are intrinsically encoded in the MRI features, a conclusion consistent with previous work demonstrating the ability of MRI-based models to predict chronological age (Franke et al., 2010; Cole and Franke, 2017).

Considering resting-state network characteristics, selected features mostly include inter-network connectivity with either the DMN, FPN or the visual network, except for three features, one of which shows intra-network connectivity of the FPN. These findings imply that the interactions between the DMN, the FPN and the visual network could be particularly important for PD-CD diagnosis. Changes in these networks are at the center of attention in the detection of cognitive deficits in PD (Rektorova et al., 2012; Amboni et al., 2015; Gorges et al., 2015; Wolters et al., 2019; Suo et al., 2022; Piramide et al., 2024).

The combined model with six features involved three RS features (DMN-3∼VIS-2, FP-4∼MOT-1, DMN-2∼VIS-2) and three GMV features (Left ACgG, Left PrG, Right Calc). Two ‘DMN-VIS’ features, being highly discriminative in the best-performing composite network highlight the importance of changes in connectivity between the two networks. These alterations may reflect impaired integration of internally directed cognitive processes with externally driven visual information, contributing to the visuospatial deficits and attentional impairments commonly seen even in prodromal stages. FPN is related to executive control. For example, one study found out that declining executive function was related to increasing static connectivity between the FPN and deep gray matter regions (Boon et al., 2020). Altered FC between the FPN and motor domain regions involving dual-task network was shown in PD (Vervoort et al., 2016). The anterior cingulate cortex is involved in control of executive functions and attention and is considered as part of the VAN (dorsal part) or the DMN. The precentral gyrus, part of the primary motor cortex, has been shown to be part of the neuronal circuit affected in PD and can be a target for transcranial magnetic stimulation (Chou et al., 2015). Finally, the right calcarine cortex, an important visual region, was also identified as a relevant structural marker. PD has been linked to specific visual deficits, including impaired contrast sensitivity, altered motion and object processing, and difficulties with complex visuospatial tasks such as spatial orientation and scene interpretation (Weil et al., 2016).

Taken together, the identified signature implicates disrupted frontoparietal control and alterations in connectivity between the DMN and the visual network as potential substrates of MoCA-defined cognitive impairment in PD.

### 4.4 ANCOVA results

Cognitive impairment in PD appears to be associated with both subtle regional atrophy and more pronounced changes in network connectivity. ANCOVA analysis revealed only minimal GMV differences between PD-CD and PD-ND groups, with a slight increase in the left ACgG and a modest decrease in the optic chiasm that did not survive correction for multiple comparisons. This pattern is consistent with observations that structural changes in early PD cognitive decline are subtle and localized (Jellinger, 2024; Seo et al., 2025). A GMV increase, as observed in some of our top SVM-selected features, may reflect compensatory neuroplastic mechanisms in early cognitive decline. Similar to prior findings, Biundo et al. reported regional cortical thickening in the precuneus and parieto-frontal regions among PD-MCI patients compared to PD-controls, suggesting brain resilience mechanisms (Biundo et al., 2013). Likewise, Reetz et al. described enhanced striatal GMV in preclinical forms of monogenetic PD, interpreted as early structural compensation to latent dopaminergic deficits (Reetz et al., 2010).

ANCOVA results for RS features show differences between groups, especially within the DMN, FPN, and visual network in PD-CD compared to PD-ND. In addition to reduced connectivity between the FPN and visual network, increased FC was found in the aforementioned networks, which may indicate a compensatory mechanism involving the recruitment of additional resources (Gorges et al., 2015; Hillary et al., 2015), or an absence of the typically negative association, especially between the DNM and other networks, which has already been demonstrated for FPN and DMN (Baggio et al., 2015).

It is important to note that the ANCOVA results are uncorrected for multiple comparisons. This limitation stems from the comprehensive, atlas-based analysis of structural and functional MRI data, which yielded many features. As such, the reported group differences should be interpreted as exploratory rather than confirmatory. No significant group differences in GMV or FC survived FDR correction after adjusting for age, sex, and TIV. These findings underscore the need for multivariate models like ML, which can detect complex, distributed patterns and subtle interactions across modalities that are inaccessible to linear models or standard statistical tests.

### 4.5 Clinical translation

The present findings underscore the potential of MRI-based ML approaches as scalable tools for early risk flagging of cognitive impairment in PD. Such imaging-derived biomarkers may serve as effective surrogates for cognitive screening, especially in contexts where the MoCA is limited by linguistic, cultural, or educational biases—limitations well documented in the literature. For instance, among low-education cohorts (mean ≤ 5 years of schooling), illiterate subjects score significantly lower on the MoCA-S than those completed primary school (16 vs. 20 out of 30) (Gómez et al., 2013), and even fully validated MoCA-H adaptations show statistically significant score differences across language groups despite adjustment for age and education (Theocharous et al., 2024). Moreover, the Movement Disorders Society cautioned against applying standard MoCA cut-offs in populations lacking literacy or cultural familiarity with the tasks (Federico et al., 2018).

The MRI data used in this study were acquired using clinical sequences—including high-resolution T1-weighted scans and resting-state echo-planar imaging. Structural MRI (T1) is a widely utilized imaging modality. Prior work demonstrated that standard structural MRI could support the early detection of neurodegenerative disorders, including Alzheimer’s disease (Davatzikos et al., 2008) and PD (Salvatore et al., 2014). Resting-state MRI protocols are currently only available in larger hospitals and reference centers. Nevertheless, it can be assumed that increasing findings from brain network research will contribute to greater clinical relevance and broader application. In addition, the duration of the recording should be between 6 and 13 min, depending on the objective and the desired reliability (Birn et al., 2013; Kumar et al., 2024). A longer recording time could also compromise the clinical applicability of the results.

Our results suggest that MRI-derived features alone—potentially even without additional clinical parameters—may be sufficient to identify PD patients at risk for cognitive decline. This supports the development of objective, non-invasive diagnostic tools that can be deployed in diverse clinical settings to identify individuals at high risk at an early stage. Moreover, the ability to longitudinally monitor imaging biomarkers offers opportunities for precision medicine, enabling personalized intervention strategies and potentially slowing cognitive deterioration. Future work should assess the utility of these markers in tracking disease progression and evaluating therapeutic efficacy (Aarsland et al., 2001; Williams-Gray et al., 2013).

### 4.6 Strengths and limitations

This study employs a comprehensive imaging protocol that combines high-resolution structural (GMV), and functional (resting-state FC) MRI data derived from clinical protocols with simple demographic and clinical parameters. In practice, not all examinations included the resting-state sequence, which slightly limits full routine applicability but reflects real-world clinical conditions. The multimodal integration nonetheless enhanced the model’s sensitivity to early cognitive deficits in PD. Additionally, robust feature selection using bootstrapping and exhaustive search identifies consistently discriminative features.

Despite these strengths, several limitations must be acknowledged. First, the small, single-center sample may introduce bias and limit the generalizability of the findings. The lack of external validation calls for testing in larger, independent samples to confirm the model’s robustness and reproducibility. Second, the education of our cohort was relatively high, possibly making it difficult to convert the findings to the general population. Third, a direct comparison with a control group was outside the scope of this study, so we did not incorporate a healthy control group. Fourth, the cross-sectional design restricts our ability to track the temporal evolution of cognitive impairment, necessitating longitudinal studies to assess progression from early deficits to overt dementia. Finally, although we employed ten-fold cross-validation to minimize overfitting, the feature selection procedure was not fully separated from the validation loop, which may introduce an optimistic bias in performance estimates (i.e., information leakage).

### 4.7 Future directions

The present study, which is an exploratory pilot study conducted in a well-defined clinical cohort, is intended to inform future research directions and to serve as a methodological and conceptual basis for validation in larger, independent and multi-center datasets. Such studies would help to reduce single-center bias and validate the model in different clinical settings. In addition, the integration of longitudinal analyses is essential; follow-up scans can determine whether early MRI markers reliably predict progression from MCI to dementia, allowing timely clinical intervention. The inclusion of additional biomarkers is promising. Complementary imaging modalities—such as positron emission tomography (PET), diffusion MRI and EEG, as well as combined approaches like Multi-Dynamic Multi-Echo (MDME) Sequences (Zheng et al., 2022)—could further elucidate the neurodegenerative processes underlying cognitive impairment in PD. Similarly, non-neuroimaging biomarkers, including genetic profiles and proteomic signatures, may capture the molecular dimensions of disease progression and improve model performance. This could allow the best feature combinations to be found to ultimately enable effective and cost-efficient detection of patients at risk of developing dementia. Furthermore, the exploration of alternative ML methods, such as deep learning architectures, ensemble techniques or hybrid models, could improve the detection of complex non-linear patterns across these diverse data sources. Together, these advances have the potential to refine and extend the predictive capabilities of our multimodal approach, paving the way for personalized therapeutic strategies and improved patient outcomes.

### 5 Conclusion

In conclusion, this study investigated cognitive deficits in PD patients, using imaging and basic clinical data collected in a hospital setting. To this end, we conducted comprehensive morphological and functional analyses of T1 and resting-state images. Our ML model used robust feature selection methods to identify imaging-derived features (e.g., GMV measures in the anterior cingulate gyrus or precuneus, and inter-network connectivity metrics involving DMN, FPN, and visual network). An SVM model yields promising classification accuracy in the identification of PD patients exhibiting mild cognitive deficits, as reflected in their MoCA performance. In univariate analysis of group differences, changes were seen in the FPN and predominantly between the DMN, FPN and visual network, which were present in high-discriminating FC features. This suggests that disruption of intrinsic networks may precede brain atrophy. Together, these advancements contribute to a better understanding of cognitive impairment in PD, aiding diagnostics and ultimately improving clinical management.

## Data Availability

The raw data supporting the conclusions of this article will be made available by the authors, without undue reservation.
