# Supplementary material for: A multimodal MRI framework employing machine learning for detecting beginning cognitive impairment in Parkinson’s disease
Source: Front Neurosci. 2025 Nov 26;19:1689302. doi: 10.3389/fnins.2025.1689302 (PMC12689920; doi:10.3389/fnins.2025.1689302)
Supplement: Supplementary file 1 [file Data_Sheet_1.pdf]

Supplementary Section

Results

Supplementary Figure 1. Model Accuracy as a Function of Feature Subset Size for Different Modalities

The figure illustrates the accuracy as a function of the number of features used for GMV (left), FC (middle), and GMV+FC (right).

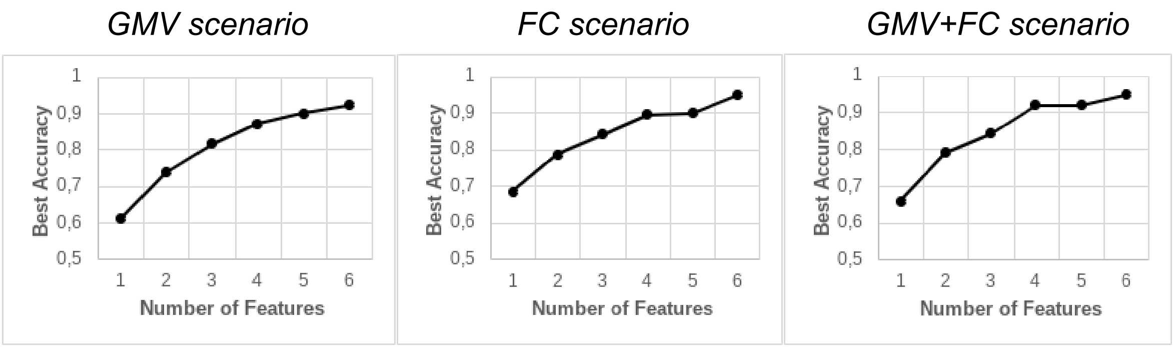

Supplementary Figure 2: Circular diagrams illustrating the selected FC features

Supplementary Figure 2 shows the main interactions between large-scale networks that best distinguish PD patients with and without cognitive deficits. On the left side the features from the FC-only scenario are shown. The features from the GMV+FC scenario are displayed on the right side. The graphs are sorted vertically from top to bottom according to the number of features from 1 to 6. Clinical and GMV features are shown in boxes adjacent to each graph. Abbreviations: Post Ins: posterior insula, ACgG: anterior cingulate gyrus, LOrG: lateral orbital gyrus, PrG: precentral gyrus, Calc: calcarine cortex.

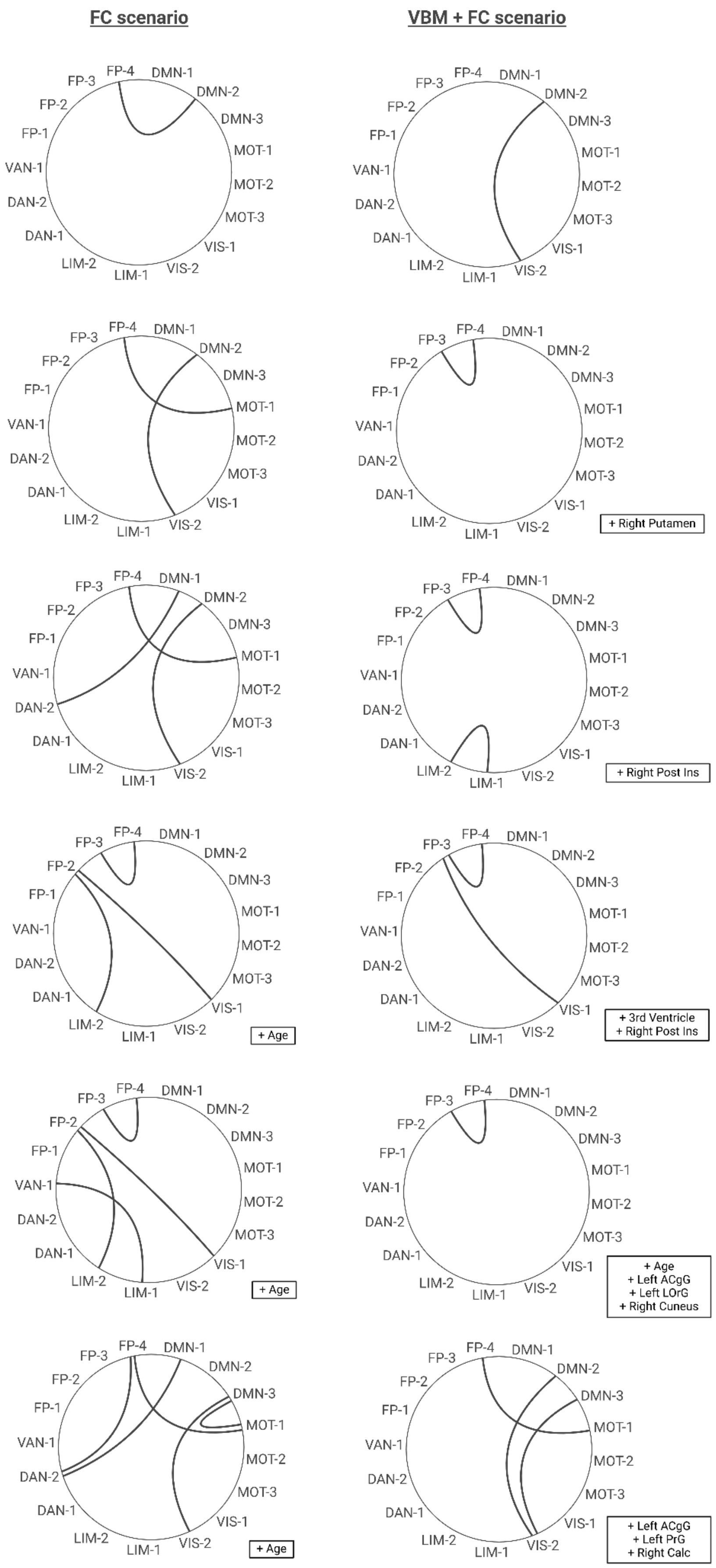
FC scenario
VBM + FC scenario
FP-1
FP-2
FP-3
FP-4
DMN-1
DMN-2
DMN-3
MOT-1
MOT-2
MOT-3
VIS-1
VIS-2
LIM-1
LIM-2
DAN-1
DAN-2
VAN-1
+ Right Putamen
+ Right Post Ins
+ Age
+ 3rd Ventricle
+ Right Post Ins
+ Age
+ Age
+ Left ACgG
+ Left LOrG
+ Right Cuneus
+ Age
+ Left ACgG
+ Left PrG
+ Right Calc
