# Supplementary material for: A multimodal MRI framework employing machine learning for detecting beginning cognitive impairment in Parkinson’s disease
Source: Front Neurosci. 2025 Nov 26;19:1689302. doi: 10.3389/fnins.2025.1689302 (PMC12689920; doi:10.3389/fnins.2025.1689302)
Supplement: Supplementary file 2 [file Supplementary_file_1.docx]

# Supplementary Section

## Methods

### Feature Reduction Algorithm

The complete Python code for the bootstrapping feature selection process is provided here. This code, integrated within the KNIME Analytics Platform, details our iterative approach to selecting features based on improvements in model performance, measured by ROC-AUC during 5-fold cross-validation. The code is fully annotated to facilitate reproducibility and further adaptation of our methodology.

| # Import necessary libraries  import knime.scripting.io as knio # KNIME I/O for reading and writing tables  import pandas as pd # Data manipulation with pandas  from sklearn.model_selection import cross_val_score # For cross-validation scoring  from sklearn.preprocessing import StandardScaler # For feature scaling (normalization)  from sklearn.svm import SVC # Support Vector Classifier  from sklearn.pipeline import make_pipeline # To build a pipeline of transforms and estimator  import random # For random feature selection  import warnings # For handling warnings  from joblib import Parallel, delayed # For parallel processing  # Suppress specific warning messages to keep the output clean  warnings.filterwarnings("ignore", category=FutureWarning)  warnings.filterwarnings("ignore", category=DeprecationWarning)  # Function to load and preprocess the data from a KNIME input table  def load_and_preprocess_data():  # Convert the first KNIME input table to a pandas DataFrame  df = knio.input_tables[0].to_pandas()  # Create the binary target variable: map 'Bin 3' to 1 (indicating cognitive deficits), others to 0  y = df.iloc[:, 0].apply(lambda x: 1 if x == 'Bin 3' else 0)  # The remaining columns are considered as features  X = df.iloc[:, 1:]  return X, y  # Function to evaluate the SVM model performance using 5-fold cross-validation  def evaluate_model(X, y, random_state=42):  # Create a pipeline with StandardScaler (to normalize data) and a linear SVM with probability estimates  model = make_pipeline(StandardScaler(), SVC(kernel='linear', probability=True, random_state=random_state))  # Compute the mean ROC-AUC score over 5 cross-validation folds  auc = cross_val_score(model, X, y, cv=5, scoring='roc_auc').mean()  return auc  # Function to perform a single bootstrap iteration for feature selection  def run_bootstrap_iteration(X, y, iteration, iterations, random_state):  # Set a unique random seed for each iteration to ensure reproducibility  random.seed(random_state + iteration)  best_features = [] # Initialize the list to store selected features for this iteration  best_auc = 0 # Initialize the best ROC-AUC achieved in this iteration  available_features = set(X.columns) # Create a set of all available features  # Loop over a fixed number of iterations to select features  for _ in range(iterations):  # If there are no more features to consider, break out of the loop  if not available_features:  break  # Randomly select a feature from the available set  new_feature = random.choice(list(available_features))  # Create a trial feature set by adding the new feature to the current best_features  trial_features = best_features + [new_feature]  # Evaluate the model performance using the trial feature set  trial_auc = evaluate_model(X[trial_features], y, random_state=random_state)  # If adding the new feature improves the ROC-AUC, update best_auc and add the feature permanently  if trial_auc > best_auc:  best_auc = trial_auc  best_features.append(new_feature)  # Remove the selected feature from the available set to avoid re-selection  available_features.remove(new_feature)  # Return the list of selected features for this bootstrap iteration  return best_features  # Function to perform parallel bootstrap feature selection across multiple iterations  def bootstrap_feature_selection_parallel(X, y, iterations=1000, bootstrap_iterations=100, random_state=42):  # Run the bootstrap iterations in parallel using all available CPU cores  all_results = Parallel(n_jobs=-1)(  delayed(run_bootstrap_iteration)(X, y, i_boot, iterations, random_state)  for i_boot in range(bootstrap_iterations)  )    # Aggregate the results: count how frequently each feature was selected across all bootstrap iterations  feature_counts = {}  for result in all_results:  for feature in result:  if feature in feature_counts:  feature_counts[feature] += 1  else:  feature_counts[feature] = 1  # Convert the aggregated feature counts into a pandas DataFrame and sort by count in descending order  feature_counts_df = pd.DataFrame(list(feature_counts.items()), columns=['Feature', 'Counts'])  feature_counts_df = feature_counts_df.sort_values(by='Counts', ascending=False)  return feature_counts_df  # Load and preprocess the data  X, y = load_and_preprocess_data()  # Execute the parallel bootstrap feature selection with specified parameters  feature_counts_df = bootstrap_feature_selection_parallel(X, y, iterations=1000, bootstrap_iterations=1000, random_state=42)  # Print the best features and their selection frequencies  print("Best features:", feature_counts_df)  # Output the resulting DataFrame to a KNIME output table  knio.output_tables[0] = knio.Table.from_pandas(feature_counts_df) |
| --- |

### SVM Classification Model

The complete Python code for the classifier is provided here. This code, integrated within the KNIME Analytics Platform, details our iterative approach to identifying the optimal feature combinations for SVM-based classification. Model performance is evaluated using 10-fold cross-validation with accuracy as the primary metric, alongside additional metrics such as precision, recall, F1 score, and ROC-AUC. The code is fully annotated to ensure reproducibility and to facilitate further adaptation of our methodology. A detailed description of the procedure is given below:

1. Data loading and preprocessing: The algorithm starts by loading the input data from KNIME. The first column of the input table is assumed to contain the target variable (e.g. class labels such as 'Bin 1' or 'Bin 2'), while the remaining columns represent the features. The data is converted into a pandas DataFrame and the features are normalized as required.
2. Generate exhaustive feature combinations: For a given subset size (ranging from 1 to a maximum number specified by the user, e.g. 5 or 6), the algorithm generates all possible combinations of features using Python's itertools.combinations. For example, if the maximum number of features is set to 6 and there are 50 candidate features, the total number of possible combinations for that subset size can be very large (e.g. 50 × 49 × 48 × 47 × 46 × 45 permutations for 6 features alone).
3. Model evaluation: Each feature combination is evaluated using a linear SVM classifier. Model performance is assessed by k-fold cross-validation (where k is user-specified, e.g. 10-fold), with accuracy as the primary metric. In addition, other metrics - including precision, recall, F1 score and ROC-AUC - are calculated. The *evaluate_combination* function trains the SVM on the selected feature subset and computes the average scores across the cross-validation folds. This evaluation is done in parallel (using joblib.Parallel) to speed up the search process over the huge number of possible feature combinations.
4. Optimal feature subset selection: For each subset size, the algorithm identifies the feature combination that achieves the highest mean accuracy. The *select_best_combination* function aggregates the evaluation results and selects the optimal set based on the primary metric (accuracy). This procedure is repeated for each feature subset size (from 1 to the user-defined maximum), resulting in a set of best-performing models corresponding to different numbers of features.
5. Results aggregation and visualization: The best results - consisting of the selected features and corresponding evaluation metrics - are aggregated into a *summary table*. In addition, a plot of best accuracy versus number of features is generated to visualize model performance trends across different feature subset sizes.

| # Import necessary libraries  import knime.scripting.io as knio # KNIME I/O module for reading/writing tables and images  import pandas as pd # For data manipulation with DataFrames  import numpy as np # For numerical operations  from sklearn.model_selection import cross_val_score # For evaluating model performance via cross-validation  from sklearn.svm import SVC # Support Vector Classifier  import matplotlib.pyplot as plt # For plotting results  import io # For handling I/O operations (e.g., saving images to a buffer)  import random # For random feature selection  import itertools # For generating combinations of features  from sklearn.metrics import make_scorer, precision_score, recall_score, f1_score # For custom scoring metrics  from sklearn.model_selection import cross_validate # For detailed cross-validation with multiple metrics  from joblib import Parallel, delayed # For parallel processing to speed up evaluation  # Load and normalize data function  def load_and_normalize_data(filepath, exclude_columns=None):  # Load the first KNIME input table and convert it to a pandas DataFrame  data = knio.input_tables[0].to_pandas()    # Extract the target variable (assumes the first column contains the target)  # Here, 'Bin 1' or 'Bin 2' (or 'Bin 3' in other contexts) indicates the class label  y = data.iloc[:, 0]    # The remaining columns are considered as features  X = data.iloc[:, 1:]    # Return features, target, and the name of the target column for reference  return X, y, data.columns[0]  # Function to select and evaluate a random set of features (not used in the main pipeline below)  def select_and_evaluate_features(X, y, num_features, iterations, k):  """Selects and evaluates a given number of features using random sampling."""  best_accuracy = 0  best_features = []  # Iterate for a specified number of iterations  for _ in range(iterations):  # Randomly sample a subset of features of size 'num_features'  selected_features = random.sample(list(X.columns), num_features)  X_selected = X[selected_features]    # Evaluate model performance using n-fold cross-validation with a linear SVM  accuracy = np.mean(cross_val_score(SVC(kernel='linear'), X_selected, y, cv=k))    # If this subset achieves better accuracy, update the best found so far  if accuracy > best_accuracy:  best_accuracy = accuracy  best_features = selected_features    return best_accuracy, best_features  # Function to evaluate a single combination of features  def evaluate_combination(X, y, combination, k, scoring):  """Evaluates a given combination of features using cross-validation and returns the average scores."""  selected_features = list(combination)  X_selected = X[selected_features]    # Evaluate the model using cross_validate to obtain scores for all specified metrics  scores = cross_validate(SVC(kernel='linear'), X_selected, y, cv=k, scoring=scoring, n_jobs=1)    # Return a dictionary with the mean score for each metric and the selected feature set  return {metric: np.mean(scores[f'test_{metric}']) for metric in scoring}, selected_features  # Function to find the best combination of a specified number of features  def select_best_combination(X, y, num_features, k, scoring, n_jobs=1):  """  Finds the best combination of features (of size 'num_features') based on the primary metric (accuracy).  It evaluates all possible combinations using parallel processing.  """  # Generate all possible combinations of features of the given size  combinations = list(itertools.combinations(X.columns, num_features))    # Evaluate all combinations in parallel  raw_results = Parallel(n_jobs=n_jobs)(  delayed(evaluate_combination)(X, y, combo, k, scoring) for combo in combinations  )  # Find the combination with the highest accuracy (primary metric)  best_result = max(raw_results, key=lambda x: x[0]['accuracy'])  best_scores, best_features = best_result  # Prepare the result dictionary with details about the best combination  result = {  'num_features': num_features,  'features': '_'.join(best_features),  'best_accuracy': best_scores['accuracy']  }  # Include other scoring metrics if necessary  for metric in scoring:  if metric != 'accuracy': # Accuracy is already included  result[f'best_{metric}'] = best_scores[metric]  return result  # MAIN CODE  # Load the data and obtain features, target, and target column name  X, y, target_column_name = load_and_normalize_data('somethingnice', exclude_columns=[])  # Retrieve parameters from a second KNIME input table (assumed to contain options)  options = knio.input_tables[1].to_pandas()  max_features = options['value'][0] # Maximum number of features to consider (e.g., 5)  k = options['value'][1] # Number of folds for cross-validation  n_jobs = -1 # Use all available CPU cores for parallel processing  # Define the scoring metrics to evaluate the model  scoring_metrics = {  'accuracy': 'accuracy',  'precision_macro': make_scorer(precision_score, average='macro', zero_division=0),  'recall_macro': make_scorer(recall_score, average='macro', zero_division=0),  'f1_macro': make_scorer(f1_score, average='macro', zero_division=0),  'roc_auc': 'roc_auc' # ROC-AUC does not require a zero_division parameter  }  # Initialize list to store the best results for each feature subset size  best_results = []  for num_features in range(1, max_features + 1):  # Find the best combination of 'num_features' features  best_result = select_best_combination(X, y, num_features, k, scoring_metrics, n_jobs)  best_results.append(best_result)  print(f"Completed evaluations for {num_features} features: Best accuracy {best_result['best_accuracy']}")    # Convert the results into a DataFrame  best_results_df = pd.DataFrame(best_results)  # Plotting the best accuracy values against the number of features  num_features_list = best_results_df['num_features']  best_accuracy_list = best_results_df['best_accuracy']  plt.figure(figsize=(10, 6))  plt.plot(num_features_list, best_accuracy_list, '-o')  plt.title(f'Best Accuracy for {target_column_name} vs. Number of Features')  plt.xlabel('Number of Features')  plt.ylabel('Best Accuracy')  plt.grid(True)  plt.tight_layout()  # Save the plot to a buffer in SVG format  buffer = io.BytesIO()  plt.savefig(buffer, format='svg')  buffer.seek(0) # Reset buffer pointer to the beginning  # Output the plot image to KNIME  knio.output_images[0] = buffer.getvalue()  # Output the best results DataFrame as a KNIME table  knio.output_tables[0] = knio.Table.from_pandas(best_results_df) |
| --- |

In our study, although the main code focuses on feature selection and model evaluation, hyperparameter tuning was performed as an additional step in the overall pipeline. This grid search process systematically varied parameters like C and any kernel-specific settings to identify the optimal configuration. The grid search itself is not explicitly shown in the provided code snippet.
