# Supplementary material for: A multimodal MRI framework employing machine learning for detecting beginning cognitive impairment in Parkinson’s disease
Source: Front Neurosci. 2025 Nov 26;19:1689302. doi: 10.3389/fnins.2025.1689302 (PMC12689920; doi:10.3389/fnins.2025.1689302)
Supplement: Supplementary file 3 [file Table_1.docx]

# Supplementary Section

## Results

### Supplementary Tables 1–3: Reduced Feature Sets

Supplementary Tables 1–3 detail the reduced feature sets for each modality-specific scenario (GMV with clinical data, FC with clinical data, and the combined GMV+FC with clinical data) obtained through our bootstrapping-based feature reduction approach. Over 1,000 iterations, the algorithm identified and retained the 50 most consistently predictive features for each scenario. These tables serve as the foundation for the subsequent exhaustive feature selection and classification analyses.

| **Supplementary table 1.** 50 Best bootstrapping features for the **GMV** scenario | | | | |  |
| --- | --- | --- | --- | --- | --- |
| # | Features | Frequency | # | Features | Frequency |
| **1**  **2**  **3**  **4**  **5**  **6**  **7**  **8**  **9**  **10**  **11**  **12**  **13**  **14**  **15**  **16**  **17**  **18**  **19**  **20**  **21**  **22**  **23**  **24**  **25** | 3rd Ventricle  Right angular gyrus  Left anterior cingulate gyrus  Left middle occipital gyrus  Age  Right MPrG  Left inferior occipital gyrus  Left calcarine cortex  Hoehn & Yahr scale  Right middle occipital gyrus  Left inferior lateral ventricle  Left precentral gyrus  Left posterior insula  Right temporal pole  Right caudate nucleus  Right precuneus  Right thalamus  Optic chiasm  Left superior frontal gyrus  Right cuneus  Right posterior insula  Left fusiform gyrus  Right putamen  Left lateral orbital gyrus  CSF | 23.5 %  23.0 %  22.6 %  21.8 %  20.1 %  20.1 %  19.0 %  18.3 %  18.3 %  18.0 %  18.0 %  17.4 %  17.1 %  17.1 %  16.9 %  16.3 %  15.5 %  15.1 %  14.9 %  14.7 %  14.4 %  14.3 %  13.8 %  13.6 %  12.6 % | **26**  **27**  **28**  **29**  **30**  **31**  **32**  **33**  **34**  **35**  **36**  **37**  **38**  **39**  **40**  **41**  **42**  **43**  **44**  **45**  **46**  **47**  **48**  **49**  **50** | Right medial orbital gyrus  Left planum temporale  Right calcarine cortex  Left MPoG  Left superior temporal gyrus  Right amygdala  Left lateral ventricle  Right superior frontal gyrus  Left Thalamus  Left SMC  Left MSFG  Right fusiform gyrus  Left precuneus  Right ventral diencephalon  Left hippocampus  UPDRS III  Left anterior insula  Right PCgG  Right inferior temporal gyrus  Right anterior orbital gyrus  Right anterior cingulate gyrus  Right postcentral gyrus  Right MPoG  Right frontal operculum  Right planum polare | 12.4 %  12.3 %  12.2 %  11.9 %  11.9 %  11.7 %  11.2 %  11.0 %  11.0 %  10.8 %  10.6 %  10.1 %  10.0 %  9.9 %  9.7 %  9.7 %  9.6 %  9.5 %  9.3 %  9.2 %  9.2 %  9.1 %  8.9 %  8.9 %  8.9 % |

Abbreviations: MPrG: precentral gyrus medial segment, CSF: cerebrospinal fluid, MPoG: postcentral gyrus medial segment, SMC: supplementary motor cortex, MSFG: superior frontal gyrus medial segment, UPDRS: Unified Parkinson’s Disease Rating Scale Part III, PCgG: posterior cingulate gyrus

| **Supplementary table 2.** 50 Best bootstrapping features for the **FC** scenario | | | | |  |
| --- | --- | --- | --- | --- | --- |
| # | Features | Frequency | # | Features | Frequency |
| **1**  **2**  **3**  **4**  **5**  **6**  **7**  **8**  **9**  **10**  **11**  **12**  **13**  **14**  **15**  **16**  **17**  **18**  **19**  **20**  **21**  **22**  **23**  **24**  **25** | DMN-3~VIS-2  DMN-2~VIS-2  FP-4~MOT-1  FP-3~FP-4  Hoehn & Yahr scale  FP-2~LIM-2  FP-2~VIS-1  FP-3~VAN-1  FP-3~LIM-2  LIM-2~MOT-1  FP-1~FP-2  DAN-2~DMN-3  VAN-1~VIS-2  Age  DAN-2~DMN-1  DAN-2~DAN1  DAN-2~MOT-1  FP-2~FP-3  LIM-1~LIM-2  LIM-1~VAN-1  FP-3~VIS-2  DMN-3~FP-4  LIM-1~MOT-1  FP-2~LIM-1  DMN-3~LIM-2 | 30.6 %  26.1 %  26.0 %  21.7 %  18.5 %  17.2 %  17.1 %  16.2 %  15.8 %  15.8 %  14.9 %  14.9 %  14.8 %  14.8 %  14.3 %  14.2 %  13.6 %  13.0 %  13.0 %  12.9 %  12.2 %  12.0 %  11.9 %  11.7 %  11.7 % | **26**  **27**  **28**  **29**  **30**  **31**  **32**  **33**  **34**  **35**  **36**  **37**  **38**  **39**  **40**  **41**  **42**  **43**  **44**  **45**  **46**  **47**  **48**  **49**  **50** | FP-2~MOT-2  LIM-2~VIS-2  DMN-3~MOT-3  VIS-1~VIS-2  FP-1~VIS-2  FP-1~MOT-2  MOT-3~VIS-2  FP-1~VAN-1  TIV  FP-3~MOT-2  FP-4~LIM-2  UPDRS III  LIM-1~VIS-1  CSF  DAN-2~MOT-2  DAN-2~FP-4  DMN-1~LIM-2  DMN-3~MOT-1  DAN-2~FP-3  MOT-1~MOT-3  LIM-1~MOT-2  DMN-2~MOT-1  DMN-1~VAN-1  LIM-2~MOT-2  DMN-2~FP-4 | 11.5 %  11.1 %  11.0 %  11.0 %  10.9 %  10.6 %  10.5 %  10.5 %  10.5 %  10.3 %  10.2 %  9.8 %  9.6 %  9.4 %  9.4 %  9.3 %  9.3 %  9.3 %  9.2 %  9.1 %  9.1 %  9.0 %  8.9 %  8.9 %  8.9 % |

Abbreviations: DMN: default mode network, VIS: visual network, FP: frontoparietal network, MOT: motor network, LIM: limbic network, VAN: ventral attention network, DAN: dorsal attention network, TIV: total intracranial volume, UPDRS III: Unified Parkinson’s Disease Rating Scale Part III, CSF: cerebrospinal fluid

| **Supplementary table 3.** 50 Best bootstrapping features for the **GMV+FC** scenario | | | | |  |
| --- | --- | --- | --- | --- | --- |
| # | Features | Frequency | # | Features | Frequency |
| **1**  **2**  **3**  **4**  **5**  **6**  **7**  **8**  **9**  **10**  **11**  **12**  **13**  **14**  **15**  **16**  **17**  **18**  **19**  **20**  **21**  **22**  **23**  **24**  **25** | DMN-3~VIS-2  FP-3~FP-4  FP-4~MOT-1  DMN-2~VIS-2  Left Inferior Lateral Ventricle  FP-2~VIS-1  Age  FP-3~VIS-2  Right angular gyrus  FP-2~LIM-2  Left anterior cingulate gyrus  Left inferior occipital gyrus  Left calcarine cortex  3rd Ventricle  Left precentral gyrus  Right Putamen  H&Y  Right posterior insula  LIM-2~MOT-1  Left middle occipital gyrus  FP-2~FP-3  FP-3~LIM-2  LIM-2~VIS-2  DAN-2~DMN-1  Left FuG fusiform gyrus | 16.8 %  16.6 %  16.2 %  15.9 %  13.3 %  12.0 %  11.5 %  11.4 %  11.4 %  11.3 %  11.3 %  11.2 %  10.3 %  10.2 %  10.2 %  10.1 %  10.0 %  10.0 %  9.7 %  8.9 %  8.8 %  8.8 %  8.7 %  8.6 %  8.5 % | **26**  **27**  **28**  **29**  **30**  **31**  **32**  **33**  **34**  **35**  **36**  **37**  **38**  **39**  **40**  **41**  **42**  **43**  **44**  **45**  **46**  **47**  **48**  **49**  **50** | Right cuneus  Right middle occipital gyrus  Left superior frontal gyrus  Left MPoG  FP-1~FP-2  Optic chiasm  Right temporal pole  Right TrIFG  LIM-1~LIM-2  DAN-2~MOT-1  Right medial orbital gyrus  DMN-3~LIM-2  Left lateral ventricle  Right precuneus  Left posterior insula  UPDRS  Right MPrG  Right amygdala  Left thalamus  DAN-2~DMN-3  FP-2~FP-4  Right thalamus  Right calcarine cortex  FP-2~MOT-2  Left lateral orbital gyrus | 8.4 %  8.4 %  8.3 %  8.3 %  8.2 %  8.2 %  8.2 %  7.9 %  7.9 %  7.9 %  7.9 %  7.8 %  7.7 %  7.6 %  7.6 %  7.6 %  7.6 %  7.6 %  7.6 %  7.5 %  7.5 %  7.3 %  7.1 %  7.1 %  7.0 % |

Abbreviations: DMN: default mode network, VIS: visual network, FP: frontoparietal network, MOT: motor network, LIM: limbic network, DAN: dorsal attention network, MPoG: postcentral gyrus medial segment, TrIFG: triangular part of the inferior frontal gyrus, MPrG: precentral gyrus medial segment

### Supplementary Table 4: ANCOVA Analysis of Imaging Features

Supplementary Table 4 provides a detailed summary of the ANCOVA results for the imaging features, with age, gender and TIV (GMV features) or age and gender (FC features) inserted as covariates. This table presents group comparisons for both grey matter volume (GMV) and functional connectivity (FC) measures, including means, standard deviations, differences between PD-CD and PD-ND groups, and p-values (both uncorrected and corrected for multiple comparisons via the Benjamini-Hochberg method). This data provides a comprehensive overview of the regional differences observed in our study.

**Supplementary table 4.** Selected Features and group comparison via ANCOVA

| Feature | PD-CD, m (SD) | PD-ND, m (SD) | Diff | F value | p (uncor.) | p (cor.) | 1 | 2 | 3 | 4 | 5 | 6 |
| --- | --- | --- | --- | --- | --- | --- | --- | --- | --- | --- | --- | --- |
| **GMV**  Left STG  Left SFG  Left Precun  Right Precun  Right MPrG  Right PCgG  Right MPoG  Right ACgG  Right AnG  Right MOrG  3rd Ventricle  Left PrG  Left ACgG  Right Cuneus  **GMV + FC**  3rd Ventricle  Left PrG  Left ACgG  Right Cuneus  Left Post Ins  Left LOrG  Right Putamen  Right Calc  FP-3~VIS-2  LIM-1~LIM-2  DMN-2~VIS-2  DMN-3~VIS-2  FP-3~FP-4  FP-4~MOT-1  **FC**  DMN-2~VIS-2  DMN-3~VIS-2  FP-3~FP-4  FP-4~MOT-1  DAN-2~DMN-1  DAN-2~FP-4  DMN-2~FP-4  DMN-3~MOT-1  FP-2~LIM-2  FP-2~VIS-1  LIM-1~VAN-1 | 5.26 (0.729)  11.25 (1.044)  8.20 (1.138)  8.73 (1.073)  1.85 (0.265)  3.07 (0.379)  0.69 (0.130)  3.18 (0.543)  7.41 (0.803)  3.37 (0.447)  0.05 (0.034)  8.82 (1.314)  3.84 (0.510)  3.46 (0.478)  0.05 (0.034)  8.82 (1.314)  3.84 (0.510)  3.46 (0.478)  1.79 (0.213)  1.77 (0.213)  3.19 (0.318)  2.24 (0.324)  -0.18 (1.269)  -0.14 (0.964)  0.33 (1.094)  0.21 (0.926)  0.34 (0.929)  -0.27 (1.052)  0.33 (1.094)  0.21 (0.926)  0.34 (0.929)  -0.27 (1.052)  -0.26 (0.897)  0.14 (0.902)  -0.18 (0.966)  -0.08 (1.124)  -0.11 (1.004)  -0.35 (0.983)  0.13 (1.089) | 5.51 (0.729)  11.41 (1.496)  8.01 (1.014)  8.41 (1.086)  1.95 (0.294)  3.01 (0.427)  0.72 (0.105)  3.08 (0.413)  7.70 (0.956)  3.42 (0.376)  0.07 (0.043)  9.21 (1.393)  3.63 (0.443)  3.25 (0.406)  0.07 (0.043)  9.21 (1.393)  3.63 (0.443)  3.25 (0.406)  1.74 (0.157)  1.82 (0.220)  3.05 (0.362)  2.13 (0.307)  0.20 (0.545)  0.16 (1.043)  -0.37 (0.753)  -0.24 (1.051)  -0.38 (0.959)  0.30 (0.870)  -0.37 (0.753)  -0.24 (1.051)  -0.38 (0.959)  0.30 (0.870)  0.29 (1.053)  -0.15 (1.105)  0.20 (1.026)  0.08 (0.866)  0.13 (1.009)  0.39 (0.887)  -0.14 (0.901) | –0.25  –0.16  0.19  0.32  –0.10  0.06  –0.03  0.10  –0.29  –0.05  –0.02  –0.39  0.21  0.21  –0.02  –0.39  0.21  0.21  0.05  –0.05  0.14  0.11  –0.38  –0.30  0.70  0.45  0.72  –0.57  0.70  0.45  0.72  –0.57  –0.55  0.29  –0.38  –0.16  –0.24  –0.74  0.27 | 0.264  0.398  0.477  2.186  1.279  0.456  0.284  0.780  0.780  0.301  0.741  0.641  4.345  3.491  0.741  0.641  4.345  3.491  1.686  0.669  1.471  1.786  1.579  1.304  4.789  4.166  5.505  1.517  4.789  4.166  5.505  1.517  3.113  1.467  1.684  0.087  2.898  4.140  0.309 | 0.611  0.533  0.494  0.149  0.266  0.504  0.598  0.383  0.384  0.587  0.396  0.429  0.045*  0.071  0.396  0.429  0.045*  0.071  0.203  0.419  0.234  0.191  0.217  0.261  0.036*  0.049*  0.025*  0.227  0.036*  0.049*  0.025*  0.227  0.087  0.234  0.203  0.770  0.098  0.050  0.582 | 1.000  1.000  1.000  1.000  1.000  1.000  1.000  1.000  1.000  1.000  1.000  1.000  1.000  1.000  1.000  1.000  1.000  1.000  1.000  1.000  1.000  1.000  1.000  1.000  1.000  1.000  1.000  1.000  1.000  1.000  1.000  1.000  1.000  1.000  1.000  1.000  1.000  1.000  1.000 | x  x  x | x  x  x  x  x  x | x  x  x  x  x  x  x  x  x | x  x  x  x  x  x  x  x  x  x  x | x  x  x  x  x  x  x  x  x  x  x  x | x  x  x  x  x  x  x  x  x  x  x  x  x  x  x  x  x |

GMV features are presented as volumes (mm³) and FC features are presented as z values (after Fisher’s r-to-z transformation). ANCOVA was performed for all 130 GMV values (with age, gender and TIV set as covariates) and all 136 FC values (with age and gender set as covariates) and presented as p value for the group. Features that were selected in both scenarios are listed twice and highlighted in grey. The columns with the numbers (1 to 6) correspond to the individual models with the corresponding number of features, with crosses indicating the presence of the feature in that model. Standard deviations (SD) are shown in parentheses. Significant results are shown with asterisk. Abbreviations: Diff: difference between mean of PD-CD and PD-ND (absolute value), uncor.: uncorrected for multiple comparisons, cor.: corrected for multiple comparisons, STG: superior temporal gyrus, SFG: superior frontal gyrus, Precun: precuneus, ACgG: anterior cingulate gyrus, MPrG: precentral gyrus medial segment, PCgG: posterior cingulate gyrus, MPoG: postcentral gyrus medial segment, AnG: angular gyrus, MOrG: medial orbital gyrus, Post Ins: posterior insula, LOrG: lateral orbital gyrus, Calc: calcarine cortex, DMN: default mode network, VIS: visual network, FP: frontoparietal network, MOT: motor network, LIM: limbic network, DAN: dorsal attention network, VAN: ventral attention network
